# Characterization of Groundnut (*Arachis hypogaea* L.) Test Locations Using Representative Testing Environments With Farmer-Preferred Traits

**DOI:** 10.3389/fpls.2021.637860

**Published:** 2021-03-15

**Authors:** Richard Oteng-Frimpong, Yussif Baba Kassim, Doris Kanvenaa Puozaa, Jerry Asalma Nboyine, Abdul-Rashid Issah, Masawudu Abdul Rasheed, Joseph Adjebeng-Danquah, Francis Kusi

**Affiliations:** Council for Scientific and Industrial Research (CSIR) – Savanna Agricultural Research Institute, Tamale, Ghana

**Keywords:** groundnut, AMMI, GGE, model diagnostics, early leaf spot, late leaf spot, multi environments trial

## Abstract

In this study, the differential rankings of 36 groundnut genotypes under varying environmental conditions were studied at various levels of phenotype. Locations that are generally accepted by the crop- and soil-based research community to represent the entire Guinea and Sudan Savanna agro-ecological zones in Ghana were characterized, this time using a crop. The characterization was done based on four farmer-preferred traits (early and late leaf spot disease ratings, and haulm and pod yields) using three models (i.e., AMMI, GGE, and Finlay–Wilkinson regression). These models were used to capture specific levels of phenotype, namely, genotype-by-environment interaction (GE), genotype main effect plus GE (G+GE), and environment and genotype main effects plus GE (E+G+GE), respectively. The effect of three major environmental covariables was also determined using factorial regression. Location main effect was found to be highly significant (*p* < 0.001), confirming its importance in cultivar placement. However, unlike genotypes where the best is usually adjudged through statistical ranking, locations are judged against a benchmark, particularly when phenotyping for disease severity. It was also found that the locations represent one complex mega-environment, justifying the need to test new technologies, including genotypes in all of them before they can be approved for adoption nationally. Again, depending on the phenotypic level considered, genotypic rankings may change, causing environmental groupings to change. For instance, all locations clustered to form one group in 2017 for early and late leaf spot diseases and pod yield when GE was considered, but the groupings changed when G+GE was considered for the same traits in the same year. As a result, assessing genotypic performance at the various levels to arrive at a consensus decision is suggested. Genotypes ICGV-IS 141120 and ICGV-IS 13937 were found to be the best performing.

## Introduction

Groundnut (*Arachis hypogaea* L.) is a leguminous oilseed crop grown in the semi-arid and subtropical regions across 40° north and south of the equator (Ajeigbe et al., 2015). In Ghana, it is the most important grain legume largely cultivated under rain-fed conditions (Oteng-Frimpong et al., 2017). The pods and haulms are important sources of income for smallholder farmers (Ajeigbe et al., 2015; Oteng-Frimpong et al., 2017). Although Africa holds the maximum global area under groundnut cultivation (i.e., 11.7 m ha representing 47.56% of total area cultivated). Meanwhile, its yield are very low (929 kg ha^−1^) compared with that of the Americas (3,632 kg ha^−1^) (Ajeigbe et al., 2015) due to a myriad of biotic and abiotic stresses (Oteng-Frimpong et al., 2015). This has resulted in a series of interventions aimed at improving pod yields on farmers' fields.

In an attempt to identify genotypes that combine inherent tolerance to biotic and abiotic stresses with high pod and haulm yields across target environments, breeders conduct multi-environment trials (METs). This has resulted in the identification of superior genotypes that are better in specific locations (specifically adapted genotypes and unstable) or across locations in a range of crops including groundnuts (Padi, 2008; Asibuo et al., 2018). Most studies, however, focus solely on the genetic component of the breeders' equation and its interaction with the environment, with little or at times no consideration for the non-genetic component.

Nonetheless, the major challenge of increasing food production by about 50% by 2050 in a context of shrinking and degraded arable land, nutrient deficiencies, increased water scarcity, uncertainty due to predicted climatic changes (Vadez et al., 2012), and under contrasting environments requires that test locations are well-delineated for the various crops to optimize crop improvement programs. For instance, a survey of groundnut fields in southern Africa found pod borers (Elaterids, Tenebrionids, doryline ants, and millipedes) to be present but rarely at sufficient densities to warrant concern (Wightman and Wightman, 1994), while doryline ants, white grubs, termites, and millipedes were a major concern on CSIR-SARI research fields in Ghana. Also, location is reported to be the key cause of variation for pod yield (tha^−1^; plot^−1^), number of pods (plot^−1^), hundred pod weight, and number of seeds (plot^−1^) in groundnut (Asibuo et al., 2018). Environmental main effect has been found to contribute the largest variation (68%) in sugar content in groundnut as compared with genotype main effect and genotype-by-environment interaction (GE), respectively (Isleib et al., 2008). In comparing the effectiveness of Eberhart and Russell joint regression method and GGE biplot analysis for GE detection, environment main effects were found to account for over 80% of all variation, compared with <20% for genotype and GE in maize hybrids (Alwala et al., 2010). This means that knowing and matching the non-genetic component of the breeders' equation (nurture) with the genetic component (nature) are key in attaining crop potential yields.

In Ghana, selection of test locations for assessing genotypes performance is largely based on agro-ecological zones. The Ghana Meteorological Agency has grouped the country into four agro-ecological zones based on the climate, namely, Coast, Forest, Transition, and North (Amekudzi et al., 2015). However, there are five, viz. tropical rain forest, semi-deciduous forest, forest-savannah transition, Guinea savannah, and Sudan savannah, in terms of vegetation. Within each ecological zone is a wide range of soil types and fertility status, cropping systems and history, diseases and pests, rainfall amount, duration and distribution, temperature, and humidity (Siaw, 2001). The interaction between these two classification scenarios (climate and vegetation) and spatial factors creates a very complex system of environments that confront groundnut cultivar development.

As a result, it is not enough to generalize and choose test locations for all crops based on climate, vegetation, or any economic consideration as has been happening in Ghana. Rather, these should be determined using the crop in question's response in all possible locations within the target environment. Representative locations can then be selected to constitute test locations for successive years, although re-characterization will be necessary after some years due to the dynamic nature of environmental conditions. Thus, the objectives of this study were to (i) categorize the target environment of CSIR-SARI groundnut breeding program into test locations using models that capture various components of the overall phenotype and (ii) determine the effect of GE on trait associations at the various levels of the phenotype.

## Materials and Methods

### Plant Materials and the Design of Experiments

Field experiments were conducted in two seasons (2017–2018) under rain-fed conditions. In the 2017 season, the experiments were conducted at Manga, Damongo, Nyankpala, and Silbele, while in the 2018 season, Tanina was added as an additional location (Supplementary File 1 Sheet 1). The locations used represent testing sites for CSIR-SARI and have been used in other studies (Marfo and Padi, 1999; Padi, 2008). The experiments comprised 36 genotypes (Supplementary File 1 Sheet 2) arranged in a lattice design and replicated twice, with six blocks per replicate. Each block contained six plots with each plot covering an area of 6.2 m^2^. Plants were spaced at 0.4 m between rows and 0.1 m within rows.

In each experimental location, fields were prepared by plowing followed by harrowing. Alligator 400 EC (pendimethalin, 400 g a.i. L^−1^) was applied immediately after planting to suppress weeds followed by one-hand weeding before pegging. Phosphorus in the form of triple super phosphate was applied as a basal fertilizer at the rate of 125 kg ha^−1^ (100 g plot^−1^) just after seedling emergence. The plants were further supplemented with calcium by the application of 400 kg ha^−1^ Omya Calciprill® (38% Ca and 0.6% Mg) between 20 and 25 days after sowing. All other recommended agronomic practices in groundnut production were adhered to. Data were collected on severity of early leaf spot (ELS) and late leaf spot (LLS) diseases as well as pod yield (PY, kg ha^−1^) in 2017. In 2018, data on above ground dry matter (ADM, kg ha^−1^) were also recorded in addition to all the datasets recorded in 2017. The ELS and LLS severity were scored on a scale of 1–9, with 1 representing complete resistance and nine representing complete defoliation of plants (Subrahmanyam et al., 1995). These scores were converted to quantitative variables using area under disease progress curve (AUDPC) based on the formula below:

$$\begin{array}{l} \text{AUDPC}=\overset{\text{a}}{\underset{\text{i}=1}{\sum }}\left[\left\{\frac{{\text{Y}}_{\text{i}}+\;{\text{Y}}_{\left(\text{i}+1\right)}}{2}\right\}\;*\;\left({\text{t}}_{\left(\text{i}+1\right)}-\;{\text{t}}_{\text{i}}\right)\right] \end{array}$$

where Y_i_ = disease level at time t_i_ and t_(i+1)_ – t_i_ = time in days between two sequential disease scores. High AUDPC value represents high disease susceptibility.

### Statistical Analysis

Normality of data was checked using the Shapiro–Wilk test. In situations where the data did not follow the Gaussian distribution, data transformation using the Box–Cox procedure (Box and Cox, 1964) with the MASS package (Ripley et al., 2019) of R statistical software (version 3.6.2) (R Core Team, 2020) was done. Data analyses were done using R statistical software based on a two-stage strategy (Malosetti et al., 2013). In the first stage, linear mixed effect model was fitted to the location specific data with restricted maximum likelihood (REML) using the lme4 package (Bates et al., 2019) of R to include all terms for design features. All model terms were regarded as random except the genotype (Equation 1).

(1) $$\begin{array}{r} {\text{y}}_{\text{ijk}}={\text{r}}_{\text{i}}+{\text{b}}_{\text{j}\left(\text{i}\right)}+{\text{g}}_{\text{k}}+\;{\text{ε}}_{\text{ijk}} \end{array}$$

where y_ijk_ is the performance of genotype k in block j nested within replicate i, r_i_ is the effect of replicate i, b_j(i)_ is the effect of block j nested within replicate i, g_k_ is the effect of genotype k, and ε_ijk_ is the residual. Significance of the fixed variables and effect estimates was tested and computed using Wald test with car (Fox et al., 2018) and lemerTest (Kuznetsova et al., 2019) packages, respectively. Estimated marginal means were computed, and multiple comparisons with Tukey's honestly significant difference (HSD) test at 0.05 probability were computed using the emmeans package (Lenth et al., 2019). The degree of freedom for the marginal means was computed based on Kenward–Roger's method, and confidence intervals were computed at 0.95.

To determine the AMMI and GGE family of models required in the second stage, model diagnosis was done using replicated data from the various locations based on signal–noise estimation (Gauch, 2013). The model diagnosis was done with an in-house algorithm designed to work in the R statistical software (Supplementary Files 2 and 3) based on the fundamental equations developed with descriptors from Gauch (2013), as shown below:

(2) $$\begin{array}{r} \text{G}{\text{E}}_{\text{N}}=\text{Erro}{\text{r}}_{\text{MS}}\times \text{G}{\text{E}}_{\text{df}} \end{array}$$

(3) $$\begin{array}{r} \text{G}{\text{E}}_{\text{S}}=\text{G}{\text{E}}_{\text{SS}}-\text{G}{\text{E}}_{\text{N}} \end{array}$$

(4) $$\begin{array}{r} \text{GG}{\text{E}}_{\text{N}}=\text{Erro}{\text{r}}_{\text{MS}}\times \text{GG}{\text{E}}_{\text{df}} \end{array}$$

(5) $$\begin{array}{r} \text{GG}{\text{E}}_{\text{S}}=\text{GG}{\text{E}}_{\text{SS}}-\text{GG}{\text{E}}_{\text{N}} \end{array}$$

where GE_N_ is the GE noise; GE_df_, GE degree of freedom; GE_S_, GE signal; GE_SS_, GE sum of squares; GGE_N_, G+GE noise; GGE_df_, G+GE degree of freedom; GGE_S_, G+GE signal; GGE_SS_, G+GE sum of squares; and Error_MS_, error mean square.

In addition to the traditional model diagnosis, the functions make the *F*-test component of the AMMI and GGE more robust by separating the Pure Error from the Error [referred to as Experimental Design in Gauch (2013)]. As a result, if blocks within environment are not statistically significant (*p* > 0.05), Error is used; otherwise, Pure Error is used in *F*-tests.

Relationship among traits at the various levels of phenotype was determined based on Pearson's correlation. The correlation analysis was done and visualized using the agricolae and corrplot (Wei et al., 2017) packages of R.

In the second stage of the analysis, the genotype-by-environment table of means was subjected to the various analytical procedures, viz. AMMI, GGE, and Finlay–Wilkinson (FW) regression. AMMI and FW regression were done using agricolae (Mendiburu, 2019) and FW (Lian, 2018) packages, respectively. GGE analysis was carried out by modifying the AMMI function (Supplementary File 3) to capture G+GE. Location-specific genotype winners from the adjusted means were used in environmental characterization (Gauch, 2013; Gauch and Moran, 2019). A factorial regression model was also fitted using the base functions in R, with mean daily precipitation, temperature, and relative humidity (RH) per growing season used as the explicit environmental covariables. The relevance of the genotype and location main effects as against the genotype-by-environment effect was determined using a linear model with genotype-by-environment mean-squares. The best-performing genotypes were selected based on results from the linear model.

## Results

### Relevance of the Environment and Genotype Main Effects Over the Genotype-by-Environment Interaction

The main effect for location was significant (*p* < 0.001) for all traits studied in 2017 and 2018 (Table 1), when compared with the GE. Manga was the location with the highest (82.9) ELS AUDPC in 2017, followed by Nyankpala (65.10) with Silbele being the lowest (37.10) (Table 2). In 2018, Manga recorded the highest AUDPC for ELS (86.90), but there was no significant difference between this location and Damongo (85.60) or Nyankpala (85.60). In contrast, Silbele and Tanina had lower AUDPC with no significant difference in this variable between these two locations. Also, Manga had the highest LLS in 2017 and 2018 (93.80 and 65.20, respectively). There was, however, no significant difference between Silbele and Tanina in 2018 (Table 2). For haulm yield, Silbele had the highest in 2017 and 2018 (3,733.00, 4,700.52), and this was significantly (*p* < 0.001) different from the other locations. Also, the haulm yield obtained in Nyankpala differed from that of Manga in both years, with Manga being among the lowest in both years. Damongo had the highest (2,272.00) pod yield in 2017, while Silbele had the lowest (Table 2). In 2018, Silbele had the highest pod yield, while Tanina had lowest (466.00). There was no significant difference between this variable in Damongo and Nyankpala.

**Table 1:** Significance of location and genotype main effects over their interaction.

	**LOC**		**GEN**		**Residuals (GE)**	
	**df**	**Sum Sq**	**df**	**Sum Sq**	**df**	**Sum Sq**
**2017**						
ELS	3	40,041^***^	35	2,487^ns^	105	7,158
LLS	3	84,536^***^	35	929^ns^	105	3,042
HYLD	3	115,996,112^***^	35	9,708,669^ns^	105	18,862,561
PYLD	3	44,757,936^***^	35	12,076,729^*^	105	20,614,700
**2018**						
ELS	4	46,903^***^	35	4,138 ^ns^	140	13,457
LLS	4	6,341.9^***^	35	2,865.9 ^ns^	140	8,598.1
HYLD	4	432,629,671^***^	35	18,038,964 ^ns^	140	1.17E+08
PYLD	4	46,143,292^***^	35	3,544,934^*^	140	11,495,647

*ELS, early leaf spot; LLS, late leaf spot; HYLD, haulm yield; PYLD, pod yield*.

* *significant at p < 0.05*,

*** *significant at *p* < 0.001; ns, not significant*.

**Table 2:** Overall genotypic performance in the various locations.

**LOC**	**ELS AUDPC**	**.group**	**LLS AUDPC**	**.group**	**HYLD (kg ha^**−1**^)**	**.group**	**PYLD (kg ha^**−1**^)**	**.group**
**2017**								
Damongo	54.00	c	37.70	c			2,272.00	a
Manga	82.90	a	93.80	a	1,304.00	c	1,764.00	b
Nyankpala	65.10	b	53.40	b	1,878.00	b	1,453.00	c
Silbele	37.10	d	31.70	d	3,733.00	a	733.00	d
**2018**								
Damongo	85.60	a	51.90	b	2,025.17	bc	867.00	b
Manga	86.90	a	65.20	a	1,401.91	d	735.00	b
Nyankpala	85.60	a	52.90	b	2,560.76	b	1,447.00	a
Silbele	55.30	b	64.60	a	4,700.52	a	1,577.00	a
Tanina	51.10	b	63.50	a	1,715.28	cd	466.00	c

*ELS, early leaf spot; LLS, late leaf spot; HYLD, haulm yield; PYLD, pod yield; AUDPC, area under disease progress curve. Values followed by dissimilar letters in the next column are significantly different (p < 0.05)*.

The genotype, ICGV-IS 141120, had the highest pod yield (2,504 kg ha^−1^) across all locations in 2017 and differed from that of 12CS-116, ICGV 86124, ICGV-IS 131091, ICGV-IS 14849, ICGV-IS 13871, and 12CS-098 (Table 3). There were no significant differences among the remaining genotypes, in terms of their pod yield (*p* ≥ 0.05). In contrast, genotype ICGV-IS 13937 had the highest pod yield (1,338 kg ha^−1^) but did not differ from that of ICGV-IS 07947, ICGV-IS 141120, and ICGV-IS 13842, while genotype ICGV-IS 131065 was the lowest in 2018 (Table 3). The lowest pod yield was recorded in ICGV-IS 131065 (690 kg ha^−1^), and there was no significant difference between this genotype and ICGV-IS 14849, ICGV-IS 131091, ICGV-IS 131051, or ICGV-IS 13984.

**Table 3:** Genotypic performance in pod yield (kg ha^−1^) across all locations.

	**2017**				**2018**			
**Genotype**	**Mean (kg ha^**−1**^)**	**Minimum**	**Maximum**	**.group**	**Mean (kg ha^**−1**^)**	**Minimum**	**Maximum**	**.group**
12CS-042	1,549	1,109	1,988	ab	784	577	990	ab
12CS-098	1,043	604	1,482	b	976	770	1,183	ab
12CS-116	1,256	817	1,695	b	896	689	1,102	ab
CHINESE	1,519	1,079	1,958	ab	869	663	1,076	ab
ICGV 86124	1,229	789	1,668	b	836	629	1,042	ab
ICGV-IS 141088	1,353	914	1,792	ab	950	743	1,156	ab
ICGV-IS 07947	2,260	1,820	2,699	ab	1,198	991	1,404	ab
ICGV-IS 09926	1,943	1,504	2,383	ab	967	761	1,174	ab
ICGV-IS 131051	1,269	830	1,708	ab	750	544	957	b
ICGV-IS 131065	1,504	1,065	1,944	ab	690	484	897	b
ICGV-IS 131090	1,406	967	1,845	ab	1,016	810	1,223	ab
ICGV-IS 131091	1,204	765	1,643	b	728	522	935	b
ICGV-IS 131096	1,638	1,198	2,077	ab	840	634	1,047	ab
ICGV-IS 13834	1,510	1,071	1,949	ab	821	614	1,027	ab
ICGV-IS 13842	1,740	1,301	2,180	ab	1,019	813	1,226	ab
ICGV-IS 13848	1,639	1,200	2,078	ab	942	735	1,148	ab
ICGV-IS 13851	1,534	1,095	1,973	ab	831	624	1,037	ab
ICGV-IS 13863	1,664	1,225	2,104	ab	889	683	1,096	ab
ICGV-IS 13864	1,680	1,241	2,119	ab	977	771	1,184	ab
ICGV-IS 13871	1,098	659	1,538	b	834	627	1,040	ab
ICGV-IS 13876	1,723	1,284	2,162	ab	927	720	1,133	ab
ICGV-IS 13910	1,426	987	1,866	ab	796	589	1,002	ab
ICGV-IS 13937	1,602	1,163	2,042	ab	1,338	1,132	1,545	a
ICGV-IS 13950	1,369	929	1,808	ab	925	719	1,132	ab
ICGV-IS 13979	1,661	1,222	2,100	ab	1,005	798	1,211	ab
ICGV-IS 13984	1,501	1,061	1,940	ab	757	550	963	b
ICGV-IS 13989	1,658	1,219	2,097	ab	893	686	1,099	ab
ICGV-IS 141120	2,504	2,065	2,943	a	1,092	885	1,298	ab
ICGV-IS 14849	1,161	721	1,600	b	722	515	928	b
ICGV-IS 14857	1,345	906	1,785	ab	930	723	1,136	ab
ICGV-IS 14876	1,821	1,381	2,260	ab	836	630	1,043	ab
ICGV-IS 14877	1,622	1,183	2,061	ab	973	766	1,179	ab
ICGV-IS 14880	1,649	1,209	2,088	ab	955	749	1,162	ab
ICGV-IS 14928	1,745	1,306	2,184	ab	909	703	1,116	ab
ICGV-IS 14943	1,702	1,263	2,141	ab	836	629	1,042	ab
YENYAWOSO	1,467	1,028	1,906	ab	914	708	1,121	ab

*Values followed by dissimilar letters in the next column are significantly different (p < 0.05)*.

### AMMI Model Diagnosis Based on Genotype-by-Environment Interaction Signal–Noise Estimation

GGE model diagnosis does not exist in any of the statistical software currently available, while the AMMI model diagnosis only exists in the AMMISOFT software (Gauch and Moran, 2019). However, a rearrangement of data is necessary if AMMISOFT is to be used. An algorithm was therefore written to capture the signal in the respective multiplicative terms of each model from the total multiplicative terms leaving the noise signal.

The environment and genotype main effects as well as the GE were statistically significant (*p* < 0.01) for all the studied traits in 2017 (Table 4). However, the AMMI model diagnosis based on Gollob's test showed AMMI3 as the appropriate model for ELS disease, while AMMI2 was the appropriate model for haulm and LLS disease, respectively, with AMMI1 being appropriate for pod yield. On the other hand, model diagnosis based on signal–noise estimation revealed AMMI1 as the appropriate model for the ELS disease and haulm yield with AMMI0 being the appropriate model for LLS disease and pod yield, respectively. The GE signal present in the overall GE for ELS and LLS diseases and haulm and pod yields data was 72.16, 53.80, 62.71, and 45.03%, respectively, with the rest being noise (Table 4). The signal (GE_S_) captured by the interactive principal components (PCs) for ELS disease and haulm yield were 71.15 and 95.79%, respectively.

**Table 4:** *F*-test of main and interaction effects, Gollob's test of multiplicative terms, and *F*_R_-test of the entire AMMI model.

		**ELS_E_1to9**		**HYLD_kgHa**		**LLS_E_1to9**		**PY_Calc_kgha**	
	**df**	**Sum Sq**	**Pr(>F)**	**Sum Sq**	**Pr(>F)**	**Sum Sq**	**Pr(>F)**	**Sum Sq**	**Pr(>F)**
**2017**									
***F*****-test**									
ENV	3	267,307	0.000^***^	56.16	0.00^**^	21,002.00	0.000^***^	369.07	0.000^***^
GEN	35	20,164	0.000^***^	3.37	0.000^***^	315.50	0.000^***^	71.57	0.000^***^
GE	105	49,332	0.000^***^	7.75	0.000^***^	788.80	0.000^***^	95.37	0.000^***^
PC1	37	25,328 (52.9%)	0.000^***^	4.66 (60.6%)	0.000^***^	430.80 (55.0%)	0.000^***^	64.18 (67.3%)	0.000^***^
PC2	35	15,056 (31.4%)	0.000^***^	3.03 (39.4%)	0.00^**^	277.10 (35.4%)	0.000^***^	17.33 (18.2%)	0.43^ns^
PC3	33	7,502 (15.7%)	0.01^*^			75.40 (9.6%)	0.92^ns^	13.86 (14.5%)	0.66^ns^
Residuals	144	18,706		4.98		600.80		74.63	
REP(ENV)	4	257	0.97^ns^	0.68	0.00^**^	118.40	0.000^***^	7.52	0.00^**^
Pure Residuals	140	18,449		4.29		482.40		67.12	
**Model diagnosis**									
GE Signal		35,596.47 (72.16%)		4.86 (62.71%)		424.35 (53.80%)		45.03 (47.22%)	
GE noise		13,735.09 (27.84%)		2.89 (37.29%)		364.43 (46.20%)		50.34 (52.78%)	
Model family		AMMI1		AMMI1		AMMI0		AMMI0	
Signal captured		25,327.70 (71.15%)		4.66 (95.79%)		0 (0%)		0 (0%)	
**2018**									
***F*****-test**									
ENV	4	10,848.00	0.000^***^	1,050.30	0.000^***^	420.79	0.01^*^	42,226	0.000^***^
GEN	35	911.90	0.051^ns^	96.75	0.000^***^	183.84	0.00^**^	4,694	0.000^***^
GE	140	2,888.40	0.15^ns^	590.14	0.000^***^	573.37	0.01^*^	13,773	0.000^***^
PC1	38	1,291.10 (45.0%)	0.00^**^	455.40 (77.2%)	0.000^***^	242.31 (42.3%)	0.000^***^	5,939 (43.1%)	0.000^***^
PC2	36	761.40 (26.50%)	0.21^ns^	58.90 (10.0%)	0.00^**^	131.98 (23.0%)	0.09^ns^	3,823 (27.8%)	0.000^***^
PC3	34	518.50 (18.1%)	0.68^ns^	52.88 (9.0%)	0.00^**^	115.39 (20.1%)	0.36^ns^	2,400 (17.4%)	0.03^*^
PC4	32	298.90 (10.4%)	0.98^ns^	22.95 (3.9%)	0.66^ns^	83.34 (14.5%)	0.56^ns^	1,610 (11.7%)	0.30^ns^
Residuals	179	3,457.20		170.87		552.66		7,933	
REP(ENV)	5	427.90	0.000^***^	28.78	0.000^***^	51.00	0.00^**^	365	0.15^ns^
Pure Residuals	174	3,029.30		142.09		501.66		7,568	
**Model diagnosis**									
GE Signal		436.96 (15.13%)		475.82 (80.63%)		169.74 (29.60%)		7,533.74 (54.70%)	
GE noise		2,451.48 (84.87%)		114.32 (19.37%)		403.64 (70.40%)		6,239.53 (45.30%)	
Model family		AMMI0		AMMI1		AMMI0		AMMI1	
Signal captured		0 (0%)		455.40 (95.71%)		0 (0%)		5,939.15 (78.83%)	
**2017 and 2018**									
***F*****-test**									
ENV	8	17,374.90	0.000^***^	73.85	0.000^***^	6,312.20	0.000^***^	217.70	0.000^***^
GEN	35	684.10	0.000^***^	3.55	0.000^***^	143.00	0.00^**^	18.67	0.000^***^
GE	280	3,954.60	0.000^***^	33.43	0.000^***^	936.50	0.000^***^	61.75	0.000^***^
PC1	42	1,047.00 (26.9%)	0.000^***^	21.10 (63.1%)	0.000^***^	282.80 (30.3%)	0.000^***^	22.87 (37.1%)	0.000^***^
PC2	40	857.60 (22.0%)	0.000^***^	3.19 (9.5%)	0.000^***^	190.80 (20.5%)	0.000^***^	13.21 (21.4%)	0.000^***^
PC3	38	545.80 (14.0%)	0.01^*^	2.81 (8.4%)	0.000^***^	162.40 (17.4%)	0.000^***^	7.94 (12.9%)	0.000^***^
PC4	36	420.10 (10.8%)	0.09^ns^	2.19 (6.5%)	0.00^**^	100.10 (10.7%)	0.09^ns^	6.67 (10.8%)	0.00^**^
Residuals	324	3,006.30		10.86		719.70		34.74	
REP(ENV)	9	308.40	0.000^***^	1.81	0.000^***^	79.00	0.000^***^	2.05	0.02^*^
Pure Residuals	315	2,697.90		9.05		640.70		32.67	
**Model diagnosis**									
GE Signal		1,533.46 (38.78%)		25.45 (76.13%)		363.37 (38.80%)		32.51 (52.64%)	
GE noise		2,421.15 (61.22%)		7.98 (23.87%)		573.16 (61.20%)		29.24 (47.36%)	
Model family		AMMI1		AMMI2		AMMI1		AMMI1	
Signal captured		1,047.03 (68.28%)		24.29 (95.44%)		282.77 (77.82%)		22.87 (70.37%)	

* *significant at p < 0.05*,

** *significant at p < 0.01*,

*** *significant at p < 0.001; ns, not significant*.

In 2018, the environment main effect was significant for all traits (Table 4). Also, that of the genotype main effect was significant for all traits, except ELS. Similarly, the GE was not significant (*p* > 0.05) for ELS, although the AMMI model diagnosis based on Gollob's test suggested AMMI1 as the appropriate model for this trait. On the other hand, AMMI3, 1, and 3 were suggested as appropriate models for haulm yield, LLS, and pod yield, respectively. However, signal–noise estimation showed AMMI0 as the appropriate model for ELS and LLS diseases, with AMMI1 being appropriate for pod and haulm yields (Table 4). Also, the total GE signal present was 15.13, 29.60, 80.63, and 54.70% for ELS and LLS diseases and haulm and pod yields, respectively. The GE_S_ captured by the PCs for haulm and pod yields were 95.71 and 78.83%, respectively.

For combined analysis of 2017 and 2018 years data, the environment and the genotype main effects as well as the GE were significant (*p* < 0.01) for all traits, with Gollob's test diagnosing AMMI3 for ELS and LLS diseases and AMMI6 and 4 for haulm and pod yields, respectively (Table 4). However, signal–noise estimation showed AMMI1 as the most appropriate model for ELS and LLS diseases and pod yield with AMMI2 as the most appropriate model for haulm yield. The GE_S_ captured by the selected PCs for ELS disease, haulm yield, LLS disease, and pod yield were 68.28, 95.44, 77.82, and 70.37%, respectively.

### GGE Model Diagnosis Based on Genotype Main Effect Plus Genotype-by-Environment Interaction Signal–Noise Estimation

The environment main effect of the GGE model was significant (*p* < 0.01) for all traits studied in 2017 (Table 5). Also, the genotype main effect plus GE (G+GE) was significant for all traits. Gollob's test showed GGE3 as the appropriate model for ELS and LLS diseases, whereas GGE2 and 1 were shown as appropriate models for haulm and pod yields, respectively. On the other hand, signal–noise estimation suggested GGE2 for ELS disease, GGE1 for haulm yield and LLS disease, and GGE0 for pod yield. The GGE_S_ was 73.65, 61.02, 56.0, and 59.80% for ELS disease, haulm yield, LLS disease, and pod yield, respectively, with the signal captured by the PCs for ELS and LLS diseases, and haulm yield being 97.12 and 74.15%, and 77.96%, respectively (Table 5).

**Table 5:** *F*-test of main and interaction effects, Gollob's test of multiplicative terms, and F_R_-test of the entire GGE model.

		**ELS_E_1to9**		**HYLD_kgHa**		**LLS_E_1to9**		**PY_Calc_kgha**	
	**df**	**Sum Sq**	**Pr(>F)**	**Sum Sq**	**Pr(>F)**	**Sum Sq**	**Pr(>F)**	**Sum Sq**	**Pr(>F)**
**2017**									
***F*****-test**									
ENV	3	267,307.00	0.000^***^	56.16	0.00^**^	21,002.00	0.000^***^	369.07	0.00^***^
GGE	140	69,496.00	0.000^***^	11.12	0.000^***^	1,104.30	0.000^***^	166.94	0.00^***^
PC1	37	34,409.00 (52.2%)	0.000^***^	5.29 (47.7%)	0.000^***^	458.50 (42.60%)	0.000^***^	117.80 (70.6%)	0.00^***^
PC2	35	15,302.00 (23.2%)	0.000^***^	3.19 (28.7%)	0.000^***^	383.20 (35.6%)	0.000^***^	21.43 (12.8%)	0.16^ns^
PC3	33	8,905.00 (13.5%)	0.00^**^			220.70 (20.5%)	0.00^**^	16.78 (10.1%)	0.39^ns^
Residuals	144	18,706.00		4.98		600.80		74.63	
REP(ENV)	4	257.00	0.75^ns^	0.68	0.00^**^	118.40	0.000^***^	7.52	0.00^**^
Pure Residuals	140	18,449.00		4.29		482.40		67.12	
**Model diagnosis**									
GGE Signal		51,182.48 (73.65%)		6.78 (61.02%)		618.42 (56.0%)		99.82 (59.80%)	
GGE noise		183.13.45 (26.35%)		4.33 (38.98%)		485.91 (44.0%)		67.12 (40.2%)	
Model family		GGE2		GGE1		GGE1		GGE0	
Signal captured		49,710.17 (97.12%)		5.29 (77.96%)		458.53 (74.15%)		0 (0%)	
**2018**									
***F*****-test**									
ENV	4	10,848.00	0.000^***^	1,050.30	0.000^***^	420.79	0.01^*^	42,226	0.000^***^
GGE	175	3,800.30	0.08^ns^	686.90	0.000^***^	757.21	0.00^**^	18,467	0.000^***^
PC1	38	1,455.60 (38.4%)	0.000^***^	489.58 (71.3%)	0.000^***^	297.54 (39.4%)	0.000^***^	6,984 (37.8%)	0.000^***^
PC2	36	958.50 (25.3%)	0.04^*^	87.09 (12.7%)	0.000^***^	187.94 (24.9%)	0.00^**^	4,515 (24.5%)	0.000^***^
PC3	34	758.30 (20.0%)	0.16^ns^	58.09 (8.5%)	0.00^**^	117.72 (15.6%)	0.22^ns^	3,774 (20.4%)	0.000^***^
PC4	32	345.90 (9.1%)	0.94^ns^	44.71 (6.5%)	0.02^*^	87.45 (11.6%)	0.55^ns^	2,043 (11.1%)	0.08^ns^
Residuals	179	3,457.20		170.87		552.66		7,933	
REP(ENV)	5	427.90	0.000^***^	28.78	0.000^***^	51.00	0.00^**^	365	0.14^ns^
Pure Residuals	174	3,029.30		142.09		501.66		7,568	
**Model diagnosis**									
GxE Signal		735.95 (19.37%)		543.99 (79.2%)		252.67 (33.37%)		10,667.64 (57.77%)	
GxE noise		3,064.35 (80.63%)		142.90 (20.8%)		504.55 (66.63%)		7,799.42 (42.23%)	
Model family		GGE0		GGE1		GGE0		GGE1	
Signal captured		0 (0%)		489.58 (90.0%)		0 (0%)		6,983.23 (65.47%)	
**2017 and 2018**									
***F*****-test**									
ENV	8	17,374.90	0.000^***^	73.85	0.000^***^	6,312.20	0.000^***^	217.70	0.000^***^
GGE	315	4,638.70	0.000^***^	36.98	0.000^***^	1,079.50	0.000^***^	80.41	0.000^***^
PC1	42	1,164.10 (25.8%)	0.000^***^	22.16 (60.0%)	0.000^***^	317.90 (29.8%)	0.000^***^	33.11 (41.2%)	0.000^***^
PC2	40	860.50 (19.1%)	0.000^***^	3.66 (9.9%)	0.000^***^	204.00 (19.1%)	0.000^***^	14.07 (17.5%)	0.000^***^
PC3	38	709.80 (15.7%)	0.000^***^	3.18 (14.2%)	0.000^***^	176.50 (16.5%)	0.000^***^	12.35 (15.4%)	0.000^***^
PC4	36	540.80 (12.0%)	0.00^**^	2.78 (7.5%)	0.000^***^	104.6 (9.8%)	0.06^ns^	6.74 (8.4%)	0.00^**^
Residuals	324	3,006.30		10.86		719.70		34.74	
REP(ENV)	9	308.40	0.000^***^	1.81	0.000^***^	79.00	0.000^***^	2.05	0.02^*^
Pure Residuals	315	2,697.90		9.05		640.70		32.69	
**Model diagnosis**									
GGE Signal		1,914.90 (41.28%)		27.86 (75.34%)		434.72 (40.27%)		47.52 (59.09%)	
GGE noise		2,723.80 (58.72%)		9.12 (24.66%)		644.80 (59.73%)		32.90 (40.91%)	
Model family		GGE1		GGE2		GGE1		GGE1	
Signal captured		1,164.14 (60.79%)		25.82 (92.68%)		317.90 (73.13%)		47.18 (99.30%)	

* *significant at p < 0.05*,

** *significant at p < 0.01*,

*** *significant at p < 0.001; ns, not significant*.

In 2018, the G+GE was not significant (*p* ≥ 0.05) for ELS diseases but was significant for haulm yield, LLS disease, and pod yield (Table 5). Gollob's *F*-test suggested GGE2 for ELS and LLS diseases, GGE4 for haulm yield and GG3 for pod yield, whereas signal–noise estimation showed GGE0 as the appropriate model for ELS and LLS diseases and GGE1 for haulm and pod yields. The overall GGE_S_ was 19.37, 79.20, 33.37, and 57.77% for ELS disease, haulm yield, LLS disease, and pod yield, respectively, with the signal captured by the PCs for haulm and pod yields being 90.0 and 65.47%.

The environment main effect and the G+GE were significant for all traits (*p* < 0.001) when the combined data from 2017 and 2018 were considered (Table 5). First four, six, three, and four PCs of the interactive component were significant (*p* < 0.05) for ELS, haulm yield, LLS, and pod yield, respectively, from the *F*-test. Hence, based on Gollob's test, GGE4, 6, 3, and 4 were appropriate for these trait analyses, respectively. However, signal–noise estimation diagnosed GGE1 for all the traits, except haulm yield, which GGE2 was diagnosed for. The GGE_S_ was 41.28, 75.34, 40.27, and 59.09% with the captured signal being 60.79, 92.68, 73.13, and 99.30% for ELS disease, haulm yield, LLS disease, and pod yield, respectively (Table 5).

### Relationship Among Test Locations and the Interaction With Genotypes Based on the Genotype-by-Environment Interaction

Damongo, Manga, and Silbele locations grouped to form a cluster of related locations with Nyankpala standing alone for ELS when GE and the location means were considered in 2017 (Tables 6, 7). However, when haulm yield was considered, Manga and Nyankpala had a common genotype winner for both GE and location means. All the locations clustered into a single group when LLS and pod yield were considered (Tables 6, 7). Genotypes ICGV-IS 09926 and ICGV-IS 13937 had the highest ELS disease severity scores (winners) in the various locations in 2017 when GE was considered, with 12CS-042 having the highest for LLS in all locations (Table 6). However, when the location means were considered, genotype CHINESE, which is always used as the susceptible check, had the highest ELS disease severity scores in three of the four locations for ELS and in all locations for LLS, respectively (Table 7). Also, genotypes ICGV 86124 and ICGV-IS 13851 were the winners for haulm yield, while 12CS-042 was the sole for pod yield in all locations when GE was considered (Table 6). On the other hand, when location means were considered, ICGV-IS 131090 and ICGV-IS 13871 had the highest haulm yield, whereas ICGV-IS 141120 won in all locations for pod yield.

**Table 6:** Environmental groups vs. genotype winners based on adjusted GE.

	**ELS**	**HYLD**	**LLS**	**PYLD**
**Environment**	**GE winner**	**GE winner**	**GE winner**	**GE winner**
**2017**				
Damongo	ICGV-IS 09926		12CS-042	12CS-042
Manga	ICGV-IS 09926	ICGV 86124	12CS-042	12CS-042
Nyankpala	ICGV-IS 13937	ICGV 86124	12CS-042	12CS-042
Silbele	ICGV-IS 09926	ICGV-IS 13851	12CS-042	12CS-042
**2018**				
Damongo	12CS-042	ICGV-IS 13989	12CS-042	YENYAWOSO
Manga	12CS-042	ICGV-IS 13989	12CS-042	YENYAWOSO
Nyankpala	12CS-042	ICGV-IS 14943	12CS-042	ICGV-IS 14849
Silbele	12CS-042	ICGV-IS 13989	12CS-042	ICGV-IS 14849
Tanina	12CS-042	ICGV-IS 13989	12CS-042	ICGV-IS 14849
**2017 and 2018**				
Damongo 2017	ICGV-IS 13834		ICGV-IS 141120	12CS-116
Damongo 2018	ICGV-IS 14849	ICGV-IS 141120	ICGV-IS 141120	ICGV-IS 07947
Manga 2017	ICGV-IS 14849	ICGV-IS 14857	ICGV-IS 141120	ICGV-IS 07947
Manga 2018	ICGV-IS 13834	ICGV-IS 14857	ICGV-IS 13834	ICGV-IS 07947
Nyankpala 2017	ICGV-IS 13834	ICGV 86124	ICGV-IS 13834	12CS-116
Nyankpala 2018	ICGV-IS 14849	ICGV-IS 14943	ICGV-IS 141120	12CS-116
Silbele 2017	ICGV-IS 14849	ICGV-IS 13979	ICGV-IS 141120	ICGV-IS 07947
Silbele 2018	ICGV-IS 13834	ICGV-IS 14877	ICGV-IS 13834	12CS-116
Tanina 2018	ICGV-IS 14849	ICGV-IS 13989	ICGV-IS 13834	12CS-116

*ELS, early leaf spot; HYLD, haulm yield; LLS, late leaf spot; PYLD, pod yield*.

**Table 7:** Environmental groups vs. genotype winners based on mean performance from adjusted GE.

	**ELS**	**HYLD**	**LLS**	**PYLD**
**Environment**	**Overall winner**	**Overall winner**	**Overall winner**	**Overall winner**
**2017**				
Damongo	CHINESE		CHINESE	ICGV-IS 141120
Manga	CHINESE	ICGV-IS 131090	CHINESE	ICGV-IS 141120
Nyankpala	ICGV-IS 13937	ICGV-IS 131090	CHINESE	ICGV-IS 141120
Silbele	CHINESE	ICGV-IS 13871	CHINESE	ICGV-IS 141120
**2018**				
Damongo	CHINESE	ICGV-IS 14877	ICGV-IS 13834	ICGV-IS 141120
Manga	CHINESE	ICGV-IS 07947	ICGV-IS 13834	ICGV-IS 13937
Nyankpala	CHINESE	ICGV-IS 14943	ICGV-IS 13834	ICGV-IS 13937
Silbele	CHINESE	ICGV-IS 07947	ICGV-IS 13834	ICGV-IS 13937
Tanina	CHINESE	ICGV-IS 07947	ICGV-IS 13834	ICGV-IS 13937
**2017 and 2018**				
Damongo 2017	CHINESE		CHINESE	ICGV-IS 13937
Damongo 2018	CHINESE	ICGV-IS 07947	ICGV-IS 141120	ICGV-IS 07947
Manga 2017	CHINESE	ICGV-IS 131090	CHINESE	ICGV-IS 07947
Manga 2018	ICGV-IS 13834	ICGV-IS 14857	CHINESE	ICGV-IS 141120
Nyankpala 2017	CHINESE	ICGV-IS 13848	CHINESE	ICGV-IS 141120
Nyankpala 2018	CHINESE	ICGV-IS 14943	ICGV-IS 141120	ICGV-IS 13937
Silbele 2017	ICGV-IS 131065	ICGV-IS 07947	ICGV-IS 141120	ICGV-IS 141120
Silbele 2018	CHINESE	ICGV-IS 131090	ICGV-IS 13834	ICGV-IS 13937
Tanina 2018	ICGV-IS 131065	ICGV-IS 131090	CHINESE	12CS-116

*ELS, early leaf spot; HYLD, haulm yield; LLS, late leaf spot; PYLD, pod yield*.

In 2018, all the locations clustered into a single group when GE and overall location means were considered for both ELS and LLS severity scores (Tables 6, 7). However, location Nyankpala stood out when haulm yield was considered. Damongo and Manga separated from the other locations for the GE of pod yield with Manga clustering with them when the overall location means were considered (Tables 6, 7). Genotype 12CS-042 had the highest ELS and LLS disease severity in all locations in 2018 when GE alone was considered. However, CHINESE and ICGV-IS 13834 were the most susceptible for ELS and LLS diseases, respectively, in all locations when the locations means were considered. Also, genotype ICGV-IS 13989 had the highest haulm yield in four of the five locations when GE was considered, while ICGV-IS 14849 had the highest pod yield in three of the five locations (Table 6). When the location means were considered, genotypes ICGV-IS 14877, ICGV-IS 07947, and ICGV-IS 14943 had the highest haulm yield in the locations, with ICGV-IS 141120 and ICGV-IS 13937 having the highest pod yield.

For combined analysis of 2017 and 2018 years' data, repeatable patterns were observed when the location means were considered for ELS disease severity with CHINESE being the most susceptible in Damongo and Nyankpala for both years, respectively (Table 7).

### Relationship Among Test Locations and the Interaction With Genotypes Based on the Genotype Main Effect Plus Genotype-by-Environment Interaction

Locations Manga and Nyankpala formed a group of common environments, with genotype ICGV-IS 13842 being the most susceptible in these locations when ELS severity was considered in 2017 (Table 8). On the other hand, Nyankpala and Silbele had a common winner for haulm yield, with Damongo, Nyankpala, and Silbele also forming a cluster of similar locations when LLS disease was considered. When pod yield was considered, all locations formed a single cluster, with 12CS-042 being the winner (Table 8).

**Table 8:** Environmental groups vs. genotype winners based on adjusted G+GE and mean performance from adjusted G+GE.

	**ELS**	**HYLD**	**LLS**	**PYLD**
**Environment**	**G+GE winner**	**G+GE winner**	**G+GE winner**	**G+GE winner**
**2017**				
Damongo	ICGV-IS 131096		YENYAWOSO	12CS-042
Manga	ICGV-IS 13842	ICGV 86124	ICGV-IS 07947	12CS-042
Nyankpala	ICGV-IS 13842	ICGV-IS 13871	YENYAWOSO	12CS-042
Silbele	ICGV-IS 141088	ICGV-IS 13871	YENYAWOSO	12CS-042
**2018**				
Damongo	12CS-042	ICGV-IS 13989	12CS-042	ICGV-IS 07947
Manga	12CS-042	ICGV-IS 13989	12CS-042	ICGV-IS 07947
Nyankpala	12CS-042	ICGV-IS 14943	12CS-042	ICGV-IS 14849
Silbele	12CS-042	ICGV-IS 13989	12CS-042	ICGV-IS 07947
Tanina	12CS-042	ICGV-IS 13989	12CS-042	ICGV-IS 14849
**2017 and 2018**				
Damongo 2017	ICGV-IS 13834		ICGV-IS 13834	ICGV-IS 141120
Damongo 2018	ICGV-IS 13834	ICGV-IS 14877	ICGV-IS 141120	ICGV-IS 07947
Manga 2017	ICGV-IS 13834	ICGV-IS 14943	ICGV-IS 141120	ICGV-IS 07947
Manga 2018	ICGV-IS 13834	ICGV-IS 131090	ICGV-IS 13834	ICGV-IS 09926
Nyankpala 2017	ICGV-IS 13834	ICGV-IS 14943	ICGV-IS 13834	ICGV-IS 07947
Nyankpala 2018	ICGV-IS 13834	ICGV-IS 14943	ICGV-IS 141120	ICGV-IS 14849
Silbele 2017	ICGV-IS 14849	ICGV-IS 131090	ICGV-IS 141120	ICGV-IS 07947
Silbele 2018	ICGV-IS 13834	ICGV-IS 131090	ICGV-IS 13834	ICGV-IS 141120
Tanina 2018	ICGV-IS 14849	ICGV-IS 141120	ICGV-IS 13834	ICGV-IS 13937

*ELS, early leaf spot; HYLD, haulm yield; LLS, late leaf spot; PYLD, pod yield*.

In 2018, all the locations considered clustered to form one group of related locations, with genotype 12CS-042 being the most susceptible when ELS and LLS disease severities were considered (Table 8). However, when haulm yield was considered, Nyankpala did not cluster with the other locations, with genotype ICGV-IS 14943 being the winner, while ICGV-IS 13989 was the winner in the other locations. Also, Nyankpala and Tanina clustered to form common environment with ICGV-IS 14849 as the winner for pod yield. ICGV-IS 07947 was the winner for the other three locations (Table 8). For combined analysis of 2017 and 2018 years' data, all environments, except Silbele in 2017 and Tanina in 2018, clustered, with ICGV-IS 13834 being the common susceptible genotype (Table 8).

### Relationship Among Test Locations and the Interaction With Genotypes Based on the Genotype and Environment Main Effects Plus Genotype-by-Environment Interaction

Locations Damongo and Silbele clustered to form a common environment, with genotype ICGV-IS 13848 being the most susceptible when ELS disease was considered in 2017 (Table 9). Again, these locations formed a cluster, with genotype YENYAWOSO as the most susceptible when LLS was considered. In both scenarios, locations Manga and Nyankpala stood out, with genotype-specific susceptibilities (Table 9). For haulm yield, Manga and Nyankpala clustered, whereas all the locations evaluated in 2017 clustered for pod yield and had genotype ICGV-IS 141120 as the winner.

**Table 9:** Environmental groups vs. genotype winners based on adjusted E+G+GE and mean performance from adjusted E+G+GE.

	**ELS**	**HYLD**	**LLS**	**PYLD**
**Environment**	**E+G+GE winner**	**E+G+GE winner**	**E+G+GE winner**	**E+G+GE winner**
**2017**				
Damongo	ICGV-IS 13848		YENYAWOSO	ICGV-IS 141120
Manga	ICGV-IS 13842	ICGV-IS 141120	ICGV-IS 13937	ICGV-IS 141120
Nyankpala	CHINESE	ICGV-IS 141120	12CS-116	ICGV-IS 141120
Silbele	ICGV-IS 13848	ICGV-IS 13871	YENYAWOSO	ICGV-IS 141120
**2018**				
Damongo	ICGV-IS 13834	ICGV-IS 13848	ICGV-IS 14943	ICGV-IS 13937
Manga	ICGV-IS 13834	ICGV-IS 14943	ICGV-IS 13834	ICGV-IS 13937
Nyankpala	ICGV-IS 13834	ICGV-IS 09926	ICGV-IS 14928	ICGV-IS 13937
Silbele	CHINESE	ICGV-IS 13876	ICGV-IS 13834	ICGV-IS 13937
Tanina	CHINESE	ICGV-IS 13848	ICGV-IS 13834	ICGV-IS 131090
**2017 and 2018**			
Damongo 2017	CHINESE		ICGV-IS 14943	ICGV-IS 141120
Damongo 2018	ICGV-IS 13834	ICGV-IS 14880	CHINESE	ICGV-IS 141120
Manga 2017	ICGV-IS 13834	ICGV-IS 13876	ICGV-IS 13937	ICGV-IS 141120
Manga 2018	ICGV-IS 13834	ICGV-IS 13848	CHINESE	ICGV-IS 141120
Nyankpala 2017	CHINESE	ICGV-IS 13876	CHINESE	ICGV-IS 141120
Nyankpala 2018	ICGV-IS 13834	ICGV-IS 13876	CHINESE	ICGV-IS 141120
Silbele 2017	ICGV-IS 131065	ICGV-IS 14943	ICGV-IS 14849	ICGV-IS 141120
Silbele 2018	CHINESE	ICGV-IS 14880	CHINESE	ICGV-IS 141120
Tanina 2018	CHINESE	ICGV-IS 13876	CHINESE	ICGV-IS 131090

*ELS, early leaf spot; HYLD, haulm yield; LLS, late leaf spot; PYLD, pod yield*.

In 2018, locations Damongo, Manga, and Nyankpala had a common most susceptible genotype (ICGV-IS 13834), with CHINESE being the most susceptible for Silbele and Tanina for ELS disease (Table 9). On the other hand, Manga, Silbele, and Tanina all had genotype ICGV-IS 13834 as the most susceptible for LLS disease. With regard to haulm yield, genotype ICGV-IS 13848 was the common winner for Damongo and Tanina, while genotype ICGV-IS 13937 won in four of the five locations (Damongo, Manga, Nyankpala, and Silbele) for pod yield (Table 9).

When 2017 and 2018 data were jointly considered, the most consistent environmental grouping was observed for pod yield, with genotype ICGV-IS 141120 winning in eight of the nine environments (Table 9).

### Influence of Environmental Covariables on Genotypic Performance and the Relationship Among Traits at Various Levels of Phenotype

Based on the factorial regression model, the environmental (locations and years combined) main effect was significant (*p* < 0.001) for all studied traits (Supplementary File 4). However, the genotype main effect was only significant for pod yield.

**Figure 1:**
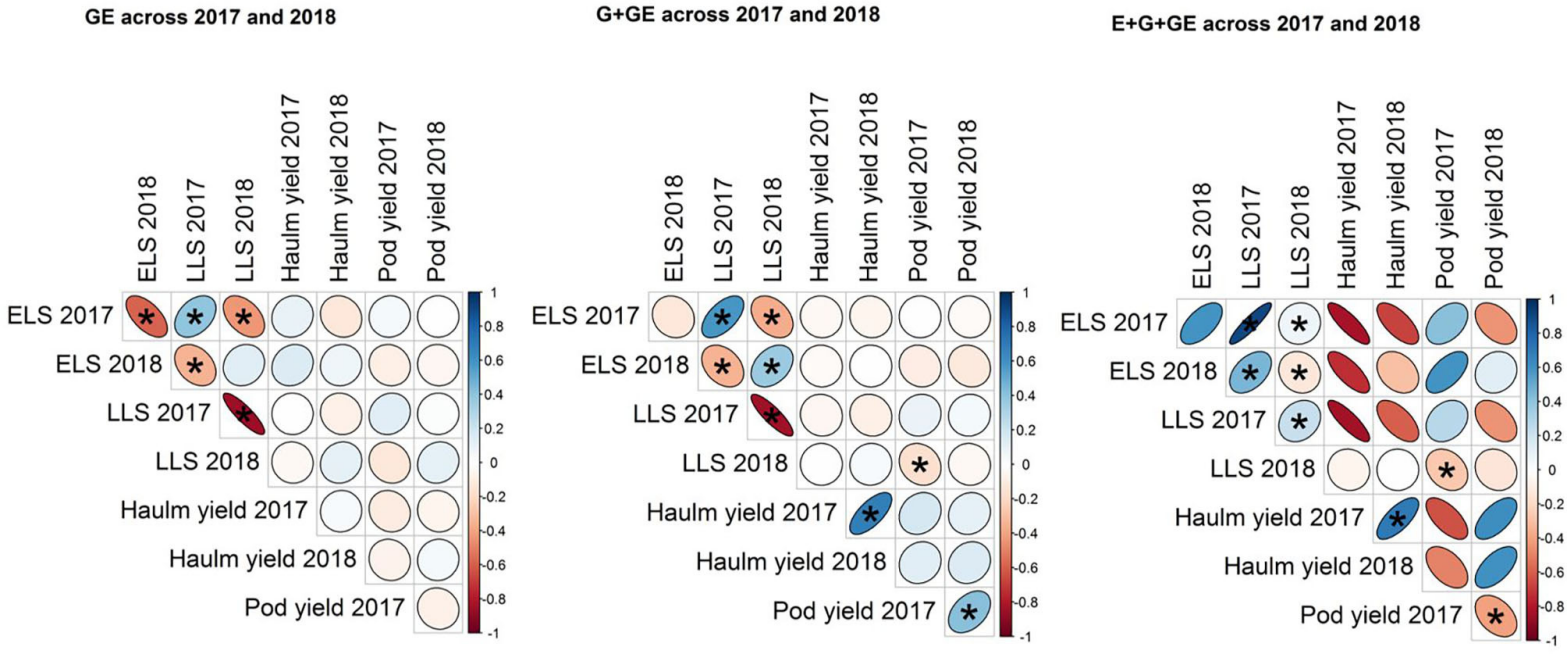
Relationship among traits at the various levels of phenotype.

ELS and LLS diseases had a significant (*p* < 0.05) positive correlation at all levels of phenotype (i.e., GE, G+GE, and E+G+GE) in all years except for E+G+GE in 2018 (Figure 1). Also, haulm in 2017 correlated positively with haulm yield in 2018 at G+GE and E+G+GE levels of phenotype. However, pod yield in 2017 correlated negatively with pod yield in 2018 at E+G+GE but positively at G+GE level of phenotype (Figure 1).

## Discussion

The present study sought to delineate locations using a crop (groundnut) with farmer-preferred traits. In this work, a high statistical significance of the location main effect for all traits in both years shows the important contribution of the non-genetic component of phenotype to overall crop performance. It also confirms the most common scenario in multi-environmental trials where the location main effect captures the largest of the total variation (Gauch, 2006). Although this component is unimportant to breeders (Gauch, 2013), it highlights the significance of putting a genotype in an optimal environment (cultivar placement) for enhanced performance. Generally, locations that ranked first in ELS and LLS disease severity (highest disease pressure) were again ranked first in pod and/or haulm yields too. This suggests that the disease condition was not too severe to affect yield. For instance, per the scale used in scoring, the highest disease severity in AUDPC that can be scored for ELS and LLS is 180 and 135, respectively. Therefore, unlike genotypes where ranking of means is required to select the best material for advancement, locations should be judged against a benchmark. This will deepen our understanding as to the amount of investment required in a location to enable a genotype perform to its maximum. Also, the genotype main effect was only significant for pod yield in both years. However, this is not surprising, because the yield obtained from pod is the end product of all the conditions and physiological processes the groundnut crop underwent during growth. It therefore highlights the importance of developing a cultivar with high genetic potential for pod yield under the set of conditions it will grow. Genotypes ICGV-IS 141120 and ICGV-IS 13937 were the best in terms of pod yield in 2017 and 2018, with average yields of 1,798 and 1,470 across years, respectively.

Traditionally, the visualization of AMMI and GGE is done using the first and second PCs (PC1 and PC2), i.e., AMMI2 and GGE2, respectively (Gauch, 2006). With the implementation of a triplot function in the agricolae package of R statistical software (Mendiburu, 2019; R Core Team, 2020), researchers now have the option of visualizing AMMI plots with three PCs on a 2D plane. However, relying on *p*-values from Gollob *F*-test to select a model family of AMMI and GGE can be misleading (Gauch, 2013; Gauch and Moran, 2019). For instance, the GE and G+GE of ELS disease in 2018 were not significant. This suggests that AMMI and GGE analyses were not justified, respectively. Meanwhile, the PC1 of the AMMI model, and PC1 and 2 of the GGE model were highly significant. Proper model diagnosis is therefore necessary in order to capture the real patterns in the data. The *F*_R_-test (Piepho, 1995) and signal–noise comparison (Gauch, 2013) are by far the most robust procedures currently available for diagnosing the AMMI models. However, the *F*_R_-test, which relies on Gollob's procedure for estimating degree of freedom (df) (Piepho, 1995; Gauch, 2013), underestimates GGE df, although its effect may be offset by declaring significance at 1 or 0.1% instead of the usual 5%. AMMI and GGE models, which were used to model phenotype at GE and G+GE levels, respectively, in the present study focused on signal–noise estimation and comparison as a means of model diagnosis, because it is highly conservative and under no circumstance does it allow some amount of noise to be captured. The downside is that it can lead to model underfitting when attention is not paid to the signal captured as seen when the suggested model captured 65.47 and 60.79% of G+GE signal in pod yield and ELS disease in 2018 and across years, respectively. However, unlike other diagnostic procedures that solely depend on statistical significance, its sensitivity is not dependent on the size of the dataset. The highest member of the AMMI model family required for any trait in this study was AMMI2, while that of GGE model was GGE2.

The use of different models (AMMI, GGE, FW regression, and factorial regression) in this study was not to determine their robustness in revealing patterns in a MET dataset. That has been discussed extensively in the literature already (Yan et al., 2000, 2001; Yan and Rajcan, 2002; Gauch, 2006, 2013; Malosetti et al., 2013; Yan, 2014). The different models were used to understand the relationship among test locations and genotypic performance at various levels of phenotype (i.e., GE, G+GE, and E+G+GE) as well as the role of explicit environmental covariables (rainfall, temperature, and RH). When the GE component was considered, patterns (similar location groupings) were repeatable across traits and across years for the same trait, although a complex scenario largely existed for across environments (location and year combined). For instance, the location groupings were the same for LLS disease and pod yield in 2017 and ELS and LLS diseases in 2018 (Tables 6, 7). The G+GE component also showed repeatable patterns across traits, across years for the same trait. In fact, across environments, GE and G+GE components of the phenotype gave similar environmental patterns and genotype winners for ELS disease severity. When the main and interactive effects (E+G+GE) were jointly considered, location groupings were again similar between ELS and LLS disease severities in 2017 and between ELS disease and pod yield in 2018, although the genotype winners were entirely different. The clustering of almost all locations (within years) and environments (across years) to form a single mega-environment among consistent patterns suggests that the locations represent one mega-environment. However, the mega-environment is a complex one (complex mega-environment) since such consistency did not cut across all traits. As a result, although these locations are testing sites and together represent two agro-ecological zones (Guinea and Sudan Savanna) (Marfo and Padi, 1999), testing in these sites approximates the entire target region. Although these locations were historically selected for trials not necessarily based on any crop's performance, their use over the years has been justified particularly for groundnut. On the other hand, the recharacterization of the entire target region may be necessary to ascertain whether these current locations will continue to be the representative environments for the respective mega-environments. However, considering the financial investment required, it will be prudent to use the on-farm approach.

Environmental components that contribute to genotype by environment in general can be categorized into two groups, i.e., those that result in predictable and those that result in unpredictable GE. An example of the predictable and unpredictable includes soil and weather parameters, respectively. Precipitation is the main source of water for groundnut cultivation in Sub-Saharan Africa, while humidity and temperature affect leaf spot disease development (Danful et al., 2019; Oteng-Frimpong and Dakora, 2019) and growth stages (Rao et al., 1992; Oteng-Frimpong et al., 2019), respectively. Hence, the lack of significance for the interactive components of the factorial regression model suggests that the predictable components of the environments were driving the genotypes by environment. It is, therefore, not surprising that clusters of location groupings were repeatable across traits and years. Also, the fact that this study yielded findings that agree to the historical characterization, which was carried out primarily based on vegetation type and other indices other than a crop performance, confirms this assertion. However, when the data from the year 2017 and 2018 were combined and analyzed, environments from the same locations (e.g., Damongo 2017 and Damongo 2018) were largely having different genotype winners resulting in them falling into different environmental groups. This scenario was observed at all levels of phenotype and indicates the present effect of latent seasonal variation, which could not be detected statistically through the factorial regression modeling. Since this has the tendency of complicating the breeder's ability to identify useful environmental patterns, it is suggested that when data from multiple years/seasons experiment are available, the analysis should be done year-wise, unless the confounding effect of the yearly variation can be properly accounted for.

Relationships among traits were consistent across the phenotypic levels of GE and G+GE for the disease-related traits. However, when the “E” component was added, the patterns changed. And since breeders are more interested in the genotype, such associations should be estimated devoid of the “E.” On the other hand, the changes in trait associations when “E” was added (i.e., E+G+GE) mean breeders ought not to ignore the environment in which cultivars are to be placed. Therefore, collaborative cultivar development that will see disciplines such as soil scientists and agronomists actively involved in the breeding process is encouraged.

## Conclusion

This study uses a comprehensive approach in test location characterization and genotype performance to examine four farmer-preferred traits with model diagnosis and at the various levels of phenotype. The study justified the use of the current test locations (Damongo, Manga, Nyankpala, Silbele, and Tanina) to represent the entire Guinea and Sudan Savanna zones in Ghana during METs. GE was driven by the predictable scenario, although latent yearly/seasonal effect exists.

Depending on the phenotypic level (i.e., GE, G+GE, or E+G+GE) at which genotypes performance are assessed, the best-performing genotype may change. Although different statistical models are used to approximate these phenotypic levels in general, comprehensively assessing genotypic performance and arriving at a decision using this approach will likely result in the identification of the most superior cultivar among a set genotypes evaluated. Considering the MET data from the 2 years independently and at the various levels of phenotype, genotypes ICGV-IS 141120 and ICGV-IS 13937 were selected and recommended for on-farm study due to their superior and consistent performance across the traits considered. Genotype CHINESE will continue to be used as a susceptible check for ELS and LLS disease studies, while ICGV-IS 13834 will be the new additional susceptible check to be added.

## Data Availability Statement

The raw data supporting the conclusions of this article will be made available by the authors, without undue reservation.

## Author Contributions

RO-F conceived the study. RO-F, DP, JA-D, and FK designed the experiments and came out with an outline. YK, JN, MR, A-RI, and FK implemented the experiments at the different locations. YK conducted the statistical analysis and produced the initial draft. RO-F and DP edited and improved the draft. All authors read and approved the final manuscript.

## Conflict of Interest

The authors declare that the research was conducted in the absence of any commercial or financial relationships that could be construed as a potential conflict of interest.
