# Supplementary material for: Characterization of Groundnut (Arachis hypogaea L.) Test Locations Using Representative Testing Environments With Farmer-Preferred Traits
Source: Front Plant Sci. 2021 Mar 15;12:637860. doi: 10.3389/fpls.2021.637860 (PMC8006269; doi:10.3389/fpls.2021.637860)
Supplement: Supplementary file 2 [file Data_Sheet_1.docx]

###############################################################################

###..AMMI model diagnosis based on signal-noise estimation and comparison..###

###############################################################################

AMMI_diag <- function(AMMIFIT){

model_aov <- AMMIFIT$ANOVA

model_ana <- AMMIFIT$analysis

model_aov1 <- AMMIFIT$ANOVA

model_ana1 <- AMMIFIT$analysis

rownames(model_aov1) <- c("ENV", "REP(ENV)", "GEN", "ENV:GEN", "Pure Residuals")

Residuals <- rowsum(model_aov1[c(2,5),], group = c("Residuals", "Residuals"), reorder = FALSE)

model_ana2 <- model_ana1[,3:7]

model_ana3 <- model_ana1[,1:2]

names(model_ana2) <- names(model_aov1)

model_aov1 <- rbind(model_aov1[c(-2,-5),], model_ana2, Residuals, model_aov1[2,], model_aov1[-1:-4,])

model_aov1[length(rownames(model_aov1))-2,3] <- model_aov1[length(rownames(model_aov1))-2,2]/model_aov1[length(rownames(model_aov1))-2,1]

BLOCKns <- function(MODELAMMIAOV){

BABS <- length(rownames(MODELAMMIAOV))

for (i in 2:(BABS-3)) {

MODELAMMIAOV[i,4] <- MODELAMMIAOV[i,3]/MODELAMMIAOV[BABS-2,3]

}

for (i in 2:(BABS-3)) {

MODELAMMIAOV[i,5] <- (1-pf(MODELAMMIAOV[i,4], MODELAMMIAOV[i,1], MODELAMMIAOV[BABS-2,1]))

}

return(MODELAMMIAOV)

}

model_aov2 <- BLOCKns(MODELAMMIAOV = model_aov1)

GxE_sig0 <- model_aov1[3,2]-(model_aov1[length(rownames(model_aov1)),3]*model_aov1[3,1])

GxE_sig1 <- model_aov2[3,2]-(model_aov2[length(rownames(model_aov2))-2,3]*model_aov2[3,1])

GxE_sig <- if(model_aov1[length(rownames(model_aov1))-1,5]<0.05){

GxE_sig0

}else{

GxE_sig1

}

row_num <- 0

XYZ <- model_ana[1:row_num,4]

{repeat {

#print(XYZ)

rm(XYZ)

row_num <- row_num+1

XYZ = sum(model_ana[1:row_num,4])

if (XYZ >= GxE_sig){

break

} else if (row_num == length(model_ana[,4])){

break

}

}

BABS1 <- length(rownames(model_aov1))

if(model_aov1[BABS1-1,5]<0.05){

cat("AMMI ")

print(model_aov1)

cat("\nF-tests use Pure Residuals because REP(Env) is significant at p<0.05 level\n")

cat("PCs")

print(model_ana3)

}else{

cat("AMMI ")

print(model_aov2)

cat("\nF-tests use Residuals because REP(Env) is not significant at p<0.05 level\n")

cat("PCs")

print(model_ana3)

}

cat("\nEstimated sums of squares for GxE signal and noise:\n")

cat("GxE total\n")

print(model_aov[4,2])

cat("GxE Signal\n")

cat(GxE_sig, "or", ((GxE_sig/model_aov[4,2])*100),"%\n")

cat("GxE noise\n")

if(model_aov1[BABS1-1,5]<0.05){

cat((model_aov1[BABS1,3]*model_aov1[3,1]), "or", (((model_aov1[BABS1,3]*model_aov1[3,1])/model_aov1[3,2])*100),"%\n")

}else{

cat((model_aov2[BABS1-2,3]*model_aov2[3,1]), "or", (((model_aov2[BABS1-2,3]*model_aov2[3,1])/model_aov2[3,2])*100),"%\n")

}

if(XYZ > GxE_sig){

cat("\nNumber of PCs required\n")

cat(row_num-1,"\n")

cat("FR-test at @ p<0.05 level diagnose AMMI",row_num-1,"\n")

cat("\nSignal captured by PCs\n")

return(cat(XYZ-model_aov1[row_num+3,2], "or", (((XYZ-model_aov1[row_num+3,2])/GxE_sig)*100), "%"))

}else{

cat("\nNumber of PCs required\n")

cat(row_num,"\n")

cat("FR-test at @ p<0.05 level diagnose AMMI",row_num,"\n")

cat("\nSignal captured by PCs\n")

return(cat(XYZ, "or", ((XYZ/GxE_sig)*100), "%"))

}

}

}

###############################################################################

###############################################################################

####...Example...####

###############################################################################

NB: install and load the agricolae package

model_0 <- with(plrv, AMMI(Locality, Genotype, Rep, Yield, console=FALSE))

AMMI_diag_diag(model_0)

###############################################################################
