# Supplementary material for: Characterization of Groundnut (Arachis hypogaea L.) Test Locations Using Representative Testing Environments With Farmer-Preferred Traits
Source: Front Plant Sci. 2021 Mar 15;12:637860. doi: 10.3389/fpls.2021.637860 (PMC8006269; doi:10.3389/fpls.2021.637860)
Supplement: Supplementary file 3 [file Data_Sheet_2.docx]

###############################################################################

####...modified AMMI function in the agricolae package for GGE analysis...####

###############################################################################

GGE_model <- function (ENV, GEN, REP, Y, MSE = 0, console = FALSE, PC = FALSE)

{

name.y <- paste(deparse(substitute(Y)))

if (console)

cat("\nANALYSIS AMMI: ", name.y, "\nClass level information\n")

ENV <- as.factor(ENV)

GEN <- as.factor(GEN)

nenv <- length(unique(ENV))

ngen <- length(unique(GEN))

if (console)

cat("\nENV: ", unique(as.character(ENV)))

if (console)

cat("\nGEN: ", unique(as.character(GEN)))

minimo <- min(ngen, nenv)

if (length(REP) > 1) {

REP <- as.factor(REP)

nrep <- length(unique(REP))

if (console)

cat("\nREP: ", unique(REP))

if (console)

cat("\n\nNumber of observations: ", length(na.omit(Y)),

"\n\n")

modelo <- aov(Y ~ ENV + REP %in% ENV + GEN + ENV:GEN)

if (console)

cat("model Y:", name.y, " ~ ENV + REP%in%ENV + GEN + ENV:GEN\n")

if (console)

cat("Random effect REP%in%ENV\n\n")

mm <- anova(modelo)

nn <- mm[2, ]

mm[2, ] <- mm[3, ]

mm[3, ] <- nn

row.names(mm)[2] <- "REP(ENV)"

row.names(mm)[3] <- "GEN "

mm[1, 4] <- mm[1, 3]/mm[2, 3]

mm[1, 5] <- 1 - pf(mm[1, 4], mm[1, 1], mm[2, 1])

if (console)

print(mm)

anova <- mm

DFE <- df.residual(modelo)

MSE <- deviance(modelo)/DFE

medy <- mean(Y, na.rm = TRUE)

if (console)

cat("\nCoeff var", "\tMean", name.y,

"\n")

if (console)

cat(sqrt(MSE) * 100/medy, "\t", medy, "\n")

}

else {

DFE <- nenv * (ngen - 1) * (REP - 1)

DFEa <- nenv * (REP - 1)

nrep <- REP

modelo <- aov(Y ~ ENV + GEN)

xx <- as.matrix(anova(modelo))

xx <- rbind(xx[1, ], xx[1, ], xx[2:3, ], xx[3, ])

row.names(xx)[4] <- "ENV:GEN"

row.names(xx)[5] <- "Residuals"

xx[2, 1] <- DFEa

xx[2, 2:5] <- NA

xx[, 2] <- xx[, 2] * nrep

xx[, 3] <- xx[, 3] * nrep

xx[5, 1] <- DFE

xx[5, 3] <- MSE

xx[5, 2] <- MSE * DFE

xx[1, 4] <- NA

if (MSE > 0)

xx[3, 4] <- xx[3, 3]/MSE

if (MSE > 0)

xx[4, 4] <- xx[4, 3]/MSE

xx[1, 5] <- NA

if (DFE > 0) {

xx[3, 5] <- 1 - pf(xx[3, 4], xx[3, 1], DFE)

xx[4, 5] <- 1 - pf(xx[4, 4], xx[4, 1], DFE)

}

row.names(xx)[1] <- "ENV "

row.names(xx)[2] <- "REP(ENV)"

if (console)

cat("\nREP: ", REP)

if (console)

cat("\n\nNumber of means: ", length(na.omit(Y)),

"\n")

if (console)

cat("\nDependent Variable:", name.y, "\n\nAnalysis of variance\n")

mm <- xx

if (console)

print(xx, na.print = "")

medy <- mean(Y, na.rm = TRUE)

if (console)

cat("\nCoeff var", "\tMean", name.y,

"\n")

if (console)

cat(sqrt(MSE) * 100/medy, "\t", medy, "\n")

}

raw <- data.frame(ENV, GEN, Y)

MEDIAS <- tapply(raw[, 3], raw[, c(1, 2)], mean)

xx <- rownames(MEDIAS)

yy <- colnames(MEDIAS)

fila <- length(xx)

col <- length(yy)

total <- fila * col

x <- character(length = total)

y <- character(length = total)

z <- numeric(length = total)

k <- 0

for (i in 1:fila) {

for (j in 1:col) {

k <- k + 1

x[k] <- xx[i]

y[k] <- yy[j]

z[k] <- MEDIAS[i, j]

}

}

MEDIAS <- data.frame(ENV = x, GEN = y, Y = z)

x <- MEDIAS[, 1]

y <- MEDIAS[, 2]

z <- MEDIAS[, 3]

modelo2 <- lm(z ~ x)

for (i in 1:length(z)) {

if (is.na(z[i]))

z[i] <- predict(modelo2, data.frame(x = MEDIAS[i,

1], y = MEDIAS[i, 2]))

}

MEDIAS <- data.frame(ENV = x, GEN = y, Y = z)

modelo1 <- lm(Y ~ ENV, data = MEDIAS)

residual <- modelo1$residuals

MEDIAS <- data.frame(MEDIAS, RESIDUAL = residual)

mlabel <- names(MEDIAS)

names(MEDIAS) <- c(mlabel[1:2], name.y, mlabel[4])

OUTRES <- MEDIAS[order(MEDIAS[, 1], MEDIAS[, 2]), ]

OUTRES2 <- by(OUTRES[, 4], OUTRES[, c(2, 1)], function(x) sum(x,

na.rm = TRUE))

OUTMED <- by(OUTRES[, 3], OUTRES[, c(2, 1)], function(x) sum(x,

na.rm = TRUE))

s <- svd(OUTRES2)

U <- s$u

L <- s$d

V <- s$v

L <- L[1:minimo]

SS <- (L^2) * nrep

SUMA <- sum(SS)

percent <- (1/SUMA) * SS * 100

DFAMMI <- rep(0, minimo)

acum <- DFAMMI

MSAMMI <- DFAMMI

F.AMMI <- DFAMMI

PROBF <- DFAMMI

acumula <- 0

for (i in 1:(minimo - 1)) {

DF <- (ngen - 1) + (nenv - 1) - (2 * i - 1)

if (DF <= 0)

break

DFAMMI[i] <- DF

acumula <- acumula + percent[i]

acum[i] <- acum[i] + acumula

MSAMMI[i] <- SS[i]/DFAMMI[i]

if (MSE > 0)

F.AMMI[i] <- round(MSAMMI[i]/MSE, 2)

else F.AMMI[i] <- NA

if (DFE > 0)

PROBF[i] <- round(1 - pf(F.AMMI[i], DFAMMI[i], DFE),

4)

else PROBF[i] <- NA

}

percent <- round(percent, 1)

acum <- round(acum, 1)

SS <- round(SS, 6)

MSAMMI <- round(MSAMMI, 6)

SSAMMI <- data.frame(percent, acum, Df = DFAMMI, `Sum Sq` = SS,

`Mean Sq` = MSAMMI, `F value` = F.AMMI, Pr.F = PROBF)

nssammi <- nrow(SSAMMI)

SSAMMI <- SSAMMI[SSAMMI$Df > 0, ]

nss <- nrow(SSAMMI)

row.names(SSAMMI) <- paste("PC", 1:nss, sep = "")

if (console) {

cat("\nAnalysis\n")

print(SSAMMI)

}

LL <- sqrt(diag(L))

SCOREG <- U %*% LL

SCOREE <- V %*% LL

SCORES <- rbind(SCOREG, SCOREE)

colnames(SCORES) <- paste("PC", 1:nssammi, sep = "")

MSCORES <- SCORES[1:ngen, ]

NSCORES <- SCORES[(ngen + 1):(ngen + nenv), ]

MGEN <- data.frame(type = "GEN", Y = apply(OUTMED,

1, mean), MSCORES)

MENV <- data.frame(type = "ENV", Y = apply(OUTMED,

2, mean), NSCORES)

bplot <- rbind(MGEN, MENV)

bplot <- bplot[, 1:(nss + 2)]

mlabel <- names(bplot)

names(bplot) <- c(mlabel[1], name.y, mlabel[c(-1, -2)])

if (minimo <= 2) {

cat("\nWarning. The analysis GGE is not possible.")

cat("\nThe number of environments and number of genotypes must be greater than 2\n")

}

if (PC)

PC <- princomp(OUTRES2, cor = FALSE)

object <- list(ANOVA = mm, GGE = OUTRES2, analysis = SSAMMI,

means = MEDIAS, biplot = bplot, PC = PC)

class(object) <- "GGE"

invisible(object)

}

###############################################################################

###############################################################################

###...GGE model diagnosis based on signal-noise estimation and comparison...###

###############################################################################

GGE_diag <- function(GGEFIT){

model_aov <- GGEFIT$ANOVA

model_ana <- GGEFIT$analysis

model_aov1 <- GGEFIT$ANOVA

model_ana1 <- GGEFIT$analysis

rownames(model_aov1) <- c("ENV", "REP(ENV)", "GEN", "ENV:GEN", "Pure Residuals")

Residuals <- rowsum(model_aov1[c(2,5),], group = c("Residuals", "Residuals"), reorder = FALSE)

model_ana2 <- model_ana1[,3:7]

model_ana3 <- model_ana1[,1:2]

names(model_ana2) <- names(model_aov1)

model_aov1 <- rbind(model_aov1[c(-2,-5),], model_ana2, Residuals, model_aov1[2,], model_aov1[-1:-4,])

gge_row <- rowsum(model_aov1[2:3,], group = c("GGE", "GGE"), reorder = FALSE)

model_aov1 <- rbind(model_aov1[1,], gge_row, model_aov1[-1:-3,])

model_aov1[,3] <- model_aov1[,2]/model_aov1[,1]

model_aov1[2,4] <- model_aov1[2,3]/model_aov1[length(rownames(model_aov1)),3]

model_aov1[2,5] <- 1-pf(model_aov1[2,4], model_aov1[2,1], model_aov1[length(rownames(model_aov1)),1])

BLOCKns <- function(MODELGGEAOV){

BABS <- length(rownames(MODELGGEAOV))

for (i in 2:(BABS-3)) {

MODELGGEAOV[i,4] <- MODELGGEAOV[i,3]/MODELGGEAOV[BABS-2,3]

}

for (i in 2:(BABS-3)) {

MODELGGEAOV[i,5] <- (1-pf(MODELGGEAOV[i,4], MODELGGEAOV[i,1], MODELGGEAOV[BABS-2,1]))

}

return(MODELGGEAOV)

}

model_aov2 <- BLOCKns(MODELGGEAOV = model_aov1)

GGE_sig0 <- model_aov1[2,2]-(model_aov1[length(rownames(model_aov1)),3]*model_aov1[2,1])

GGE_sig1 <- model_aov2[2,2]-(model_aov2[length(rownames(model_aov2))-2,3]*model_aov2[2,1])

GGE_sig <- if(model_aov1[length(rownames(model_aov1))-1,5]<0.05){

GGE_sig0

}else{

GGE_sig1

}

row_num <- 0

XYZ <- model_ana[1:row_num,4]

{repeat {

#print(XYZ)

rm(XYZ)

row_num <- row_num+1

XYZ = sum(model_ana[1:row_num,4])

if (XYZ >= GGE_sig){

break

} else if (row_num == length(model_ana[,4])){

break

}

}

BABS1 <- length(rownames(model_aov1))

if(model_aov1[BABS1-1,5]<0.05){

cat("GGE ")

print(model_aov1)

cat("\nF-tests use Pure Residuals because REP(Env) is significant at p<0.05 level\n")

cat("PCs")

print(model_ana3)

}else{

cat("GGE ")

print(model_aov2)

cat("\nF-tests use Residuals because REP(Env) is not significant at p<0.05 level\n")

cat("PCs")

print(model_ana3)

}

cat("\nEstimated sums of squares for GGE signal and noise:\n")

cat("GGE total\n")

print(gge_row[,2])

cat("GGE Signal\n")

cat(GGE_sig, "or", ((GGE_sig/gge_row[,2])*100),"%\n")

cat("GGE noise\n")

if(model_aov1[BABS1-1,5]<0.05){

cat((model_aov1[BABS1,3]*model_aov1[2,1]), "or", (((model_aov1[BABS1,3]*model_aov1[2,1])/model_aov1[2,2])*100),"%\n")

}else{

cat((model_aov2[BABS1-2,3]*model_aov2[2,1]), "or", (((model_aov2[BABS1-2,3]*model_aov2[2,1])/model_aov2[2,2])*100),"%\n")

}

if(XYZ > GGE_sig){

cat("\nNumber of PCs required\n")

cat(row_num-1,"\n")

cat("FR-test at @ p<0.05 level diagnose GGE",row_num-1,"\n")

cat("\nSignal captured by PCs\n")

return(cat(XYZ-model_aov1[row_num+2,2], "or", (((XYZ-model_aov1[row_num+2,2])/GGE_sig)*100), "%"))

}else{

cat("\nNumber of PCs required\n")

cat(row_num,"\n")

cat("FR-test at @ p<0.05 level diagnose GGE",row_num,"\n")

cat("\nSignal captured by PCs\n")

return(cat(XYZ, "or", ((XYZ/GGE_sig)*100), "%"))

}

}

}

###############################################################################

###############################################################################

####...Example...####

###############################################################################

model_1 <- with(plrv, GGE_model(Locality, Genotype, Rep, Yield, console=FALSE))

GGE_diag(model_1)

###############################################################################
