# Supplementary material for: Characterization of Groundnut (Arachis hypogaea L.) Test Locations Using Representative Testing Environments With Farmer-Preferred Traits
Source: Front Plant Sci. 2021 Mar 15;12:637860. doi: 10.3389/fpls.2021.637860 (PMC8006269; doi:10.3389/fpls.2021.637860)
Supplement: Supplementary file 4 [file Data_Sheet_3.docx]

###############################################################################

Analysis of Variance Table

Response: ELS disease

Df Sum Sq Mean Sq F value Pr(>F)

ENV 8 47416 5927.0 145.0622 < 2e-16 ***

GEN 35 1802 51.5 1.2603 0.16817

GEN:Weather_precip 35 1109 31.7 0.7752 0.81157

GEN:Weather_rh 35 1069 30.5 0.7476 0.84459

GEN:Weather_temp 35 1979 56.5 1.3836 0.09031 .

Residuals 175 7150 40.9

---

Signif. codes: 0 ‘***’ 0.001 ‘**’ 0.01 ‘*’ 0.05 ‘.’ 0.1 ‘ ’ 1

###############################################################################

###############################################################################

Analysis of Variance Table

Response: Haulm yield

Df Sum Sq Mean Sq F value Pr(>F)

ENV 7 19.0195 2.71707 59.2677 <2e-16 ***

GEN 35 1.0027 0.02865 0.6249 0.9469

GEN:Weather_precip 35 0.8729 0.02494 0.5440 0.9811

GEN:Weather_rh 35 0.6758 0.01931 0.4212 0.9981

GEN:Weather_temp 35 1.0500 0.03000 0.6544 0.9277

Residuals 139 6.3723 0.04584

---

Signif. codes: 0 ‘***’ 0.001 ‘**’ 0.01 ‘*’ 0.05 ‘.’ 0.1 ‘ ’ 1

###############################################################################

###############################################################################

Analysis of Variance Table

Response: LLS disease

Df Sum Sq Mean Sq F value Pr(>F)

ENV 8 2302.58 287.823 206.1052 <2e-16 ***

GEN 35 44.59 1.274 0.9123 0.6131

GEN:Weather_precip 35 51.99 1.485 1.0637 0.3839

GEN:Weather_rh 35 20.67 0.590 0.4228 0.9982

GEN:Weather_temp 35 53.38 1.525 1.0921 0.3457

Residuals 175 244.38 1.396

---

Signif. codes: 0 ‘***’ 0.001 ‘**’ 0.01 ‘*’ 0.05 ‘.’ 0.1 ‘ ’ 1

###############################################################################

###############################################################################

Analysis of Variance Table

Response: Pod yield

Df Sum Sq Mean Sq F value Pr(>F)

ENV 8 3044.47 380.56 126.4676 < 2.2e-16 ***

GEN 35 265.34 7.58 2.5193 4.426e-05 ***

GEN:Weather_precip 35 115.20 3.29 1.0938 0.3437

GEN:Weather_rh 35 117.08 3.35 1.1117 0.3208

GEN:Weather_temp 35 89.48 2.56 0.8496 0.7091

Residuals 173 520.58 3.01

---

Signif. codes: 0 ‘***’ 0.001 ‘**’ 0.01 ‘*’ 0.05 ‘.’ 0.1 ‘ ’ 1

###############################################################################
